# Factors associated with surgical resection in patients with Crohn’s disease: long-term evaluation

**DOI:** 10.1590/acb391924

**Published:** 2024-04-15

**Authors:** Sandro da Costa Ferreira, Lílian Rose Otoboni Aprile, Rogério Serafim Parra, Marley Ribeiro Feitosa, Patrícia Picardi Morais de Castro, Gleici de Castro da Silva Perdoná, Omar Feres, José Joaquim Ribeiro da Rocha, Luiz Ernesto de Almeida Troncon

**Affiliations:** 1 Universidade de São Paulo – Medical School – Department of Medicine – Ribeirão Preto (SP), Brazil.; 2 Universidade de São Paulo – Medical School – Department of Surgery and Anatomy – Ribeirão Preto (SP), Brazil.; 3 Universidade de São Paulo – Medical School – Department of Social Medicine – Ribeirão Preto (SP), Brazil.

**Keywords:** Crohn Disease, Smoking, General Surgery

## Abstract

**Purpose:**

To evaluate patient characteristics and factors associated with surgical resection in patients with Crohn’s disease (CD).

**Methods:**

An analysis was performed on data from 295 patients with CD in follow-up from 2001 to 2018. Medical record data comprised age, gender, location, behavior and duration of the CD, smoking, and extraintestinal manifestation. Patients were divided into two groups according to the presence or absence of surgical resection.

**Results:**

Out of the 295 patients with CD, 155 underwent surgical resection (53.2% male, mean age: 43.88 ± 14.35 years). The main indications for surgery were stenosis (44.5%), clinical intractability (15.5%), and intra-abdominal fistulas (15.5%). Smoking ( *p* < 0.001), longer CD duration ( *p* < 0.0001), ileo-colonic location ( *p* = 0.003), stenosing behavior ( *p* < 0.0001), and fistulizing behavior (*p* < 0.0001) were significantly associated with surgical resection. Initial use of biological was significantly more frequent in the group of patients without surgical resection ( *p* < 0.001).

**Conclusions:**

Patients with CD still frequently need surgical treatment. Smoking (current or past), longer disease time, stenosing and fistulizing behavior, and ileo-colonic localization in CD patients were associated with a higher risk of surgery. Awareness about factors associated with unfavorable outcome allows such patients to be treated more appropriately.

## Introduction

Crohn’s disease (CD) is a chronic, recurrent inflammatory bowel disease (IBD) that is characterized by segmental, asymmetric, and transmural inflammation. It affects any part of the gastrointestinal tract, but most commonly the terminal ileum, colon, or both^1^^,^ 2. CD has a progressive course, and its natural history is characterized by periods of clinical remission alternating with relapses^1^^,^ 2. The clinical presentation is heterogeneous, sometimes insidious, and depends on the location, severity of inflammation, and disease behavior^2^.

The symptoms presented are variable and may include diarrhea, abdominal pain, weight loss, nausea, and vomiting. Fatigue and anorexia are common symptoms, and approximately a third of patients present perianal disease^1^^,^ 2. Worsening quality of life, work productivity impairment, and an increased number of surgeries and hospitalizations have been related to CD, especially in patients with moderate to severe disease activity^3^.

At the time of diagnosis, most patients have an inflammatory phenotype, but during the disease progression to the stenosing or penetrating phenotypes may occur. This results in complications such as strictures, fistulas, or abscesses. In most cases, such patients may need surgical treatment because of these complications^1^^,^ 2 ^,^ 4.

The objective of clinical therapy in CD is to achieve a deep and prolonged remission free of steroids, preventing complications and surgeries, and interrupting the progressive course of the disease^1^^,^ 2 ^,^ 5. Until recently, therapeutic options were limited to immunosuppressive agents (thiopurines and methotrexate), and tumor necrosis factor (anti-TNF) antagonists^6^. More recently, drugs with new mechanisms of action have been incorporated into the therapeutic arsenal for the management of CD, including a selective intestinal anti-integrin (α4β7) and a monoclonal antibody against interleukin (IL)-12 and IL-23^7^^,^ 8.

Despite of how much drug treatment has advanced, most patients with CD will undergo at least one surgical resection over the course of the disease after diagnosis^9^^,^ 10. Surgical treatment is indicated in cases of failure or side effects of drug therapy, strictures with recurrent obstructive symptoms, malnutrition, perianal disease with complex fistulas, steroid dependence, dysplasia or cancer, and septic complications, such as perforations and abscesses^9^^–^ 11. It is important to note that the decision and the time to perform the surgical approach must be discussed in a multidisciplinary environment and together with the patient. The surgical technique and the exact surgical procedure depend on the indication underlying the surgery and the experience of the surgical team^9^^,^ 11 ^–^ 13.

Since most patients with CD undergo at least one intestinal resection during their lifetime, it is important to understand the main factors associated with the increased risk of surgical resection. Our study describes 17 years of experience in a single Brazilian center and provides important information on general and specific aspects of CD patients undergoing surgical resection. Thus, the aim of the present study was to evaluate the associated factors of surgical resection in patients with CD who were followed up at the university hospital.

## Methods

### Study design

This study was descriptive. A total of 690 patients were diagnosed with IBD in follow-up at the university hospital. Of these, we included 295 patients with an established diagnosis of CD between January 2001 and December 2018. We excluded patients who had a diagnosis of ulcerative colitis or undetermined IBD. We also excluded patients in which surgical treatment was involved for only perianal lesions of CD.

The diagnosis of CD was performed based on clinical, endoscopic, radiological, and histopathological aspects, and classification was performed according to the Montreal Classification^14^. Diagnoses of CD established at the time of surgery without prior medical treatment were considered urgent surgical treatment. Clinical data and descriptive statistics of the CD outpatients were obtained and analyzed through the review of medical records from the IBD registry of the university hospital. Present age, age at diagnosis, gender, ethnicity, smoking habits, the presence of extraintestinal manifestations (EIM), CD diagnosis time, location, behavior of CD, therapies, and number and type of surgeries were registered at every appointment of each patient.

Differences in demographic and clinical characteristics and predictors of surgical resection for CD were analyzed statistically. Among the therapies, corticosteroid, immunosuppressive, and biological therapies were collected for statistical analysis. Initial use of biological therapies was defined as use for up to one year after the diagnosis of CD. Surgery from the time of diagnosis and during follow-up was defined as any intra-abdominal surgical procedure for active CD (enterectomy, ileocolectomy, and colectomy). Thus, drainage of perianal abscesses and simple perianal fistulectomy were not considered as surgery in this outcome definition.

This study was approved by the local institutional research ethics committee (protocol no. 3,147/2019). All patients agreed to participate in the study, and informed consent was obtained from each patient included in the study. All procedures were in accordance with the ethical standards of the committee responsible for human experimentation (institutional and national).

### Statistical analysis

Statistical analyses were performed with the statistical software Statistical Package for the Social Sciences (SPSS) version 22 (SPSS Inc., Chicago, IL, United States of America). Data are presented as absolute numbers and percentages and means ± standard deviations (SD). Comparisons between subgroups were performed using a t-test for independent samples. To compare groups for independent samples and categorical variables, we used the χ^2^ test or Fisher’s exact test when appropriate. Survival analysis was performed using Kaplan−Meier analysis based on the log-rank test. In the multivariate model, the binary logistic regression was used. Statistical significance was set at *p* < 0.05.

## Results

### Patient characteristics

Among the 295 patients diagnosed with CD included in the study, 157 (53.2%) were male. The mean age was 43.88 ± 14.35 years, and the duration of follow-up was 13.60 ± 8.74 years. There were 248 white patients (84.1%) and 89 patients (30.2%) who were active or former smokers. There were 33 patients (11.2%) with EIM, which was mainly articular (peripheral arthritis, sacroiliitis, and ankylosing spondylitis), but there were also cutaneous cases (erythema nodosum and gangrenous pyoderma) and hepatobiliary cases (primary sclerosing cholangitis). Demographic characteristics of these patients are shown in Table 1.

**Table 1:** Baseline demographic and clinical characteristics patients with Crohn’s disease.

Variable	CD patients (n = 295)	
Age (mean ± standard deviation)	43.88 ± 14.35	
	**n**	**%**
**Gender**		
Male	157	53.2
Female	138	46.8
**Skin color**		
White	248	84.1
Mixed race	27	9.1
Black	20	6.8
**Age at the time of inflammatory bowel disease diagnosis (years old)†**		
< 16	31	10.5
16–40	203	68.8
> 40	61	20.7
**Location of Crohn’s disease†**		
Ileal	80	27.1
Colonic	34	11.5
Ileo-colonic	174	59.0
Upper gastrointestinal tract	7	2.4
**Behavior of Crohn’s disease†**		
Inflammatory	61	20.7
Stenosing	110	37.3
Fistulizing	124	42.0
Perianal disease	99	33.6
**Smoking**		
Yes	89	30.2
No	206	69.8
**Extraintestinal manifestation†**		
Yes	33	11.2
No	262	88.8
**Surgical resection**		
Yes	155	52.5
No	140	47.5

† Involvement of other parts of the body, such as joints, eyes, skin, liver and bile ducts, in patients with inflammatory bowel diseases (ulcerative colitis and Crohn’s disease).

Source: Elaborated by the authors.

**Figure 1:**
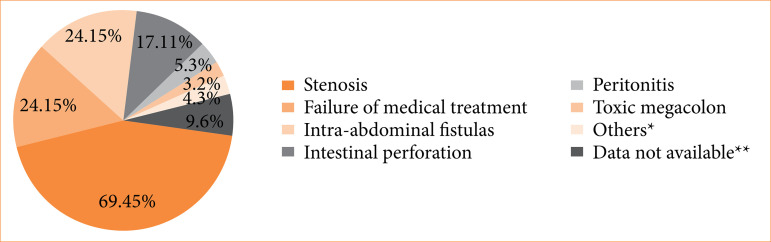
The main causes of surgical treatment among the 155 patients with Crohn’s disease who underwent surgical resection.

Throughout follow-up, 155 (52.5%) patients underwent surgery [115 enterectomies (38.9%), 73 colectomies (24.7%), and 31 (10.1%) ileocolectomies] at some point. There were 41 patients (13.9%) who underwent two surgical resections and 11 (3.7%) who underwent three or more surgical resections over the follow-up period. The main indications for surgical treatment were stenosis, in 69 patients (44.5%), followed by failure of medical treatment in 24 patients (15.5%), and intra-abdominal fistulas in 24 patients (15.5%), as shown in Fig. 1. There were 32 patients (20.6%) who had a diagnosis of CD established at the time of surgery without prior medical treatment. Comparisons regarding the baseline clinical and demographic characteristics of patients with and without surgical resection are described in Table 2.

*Others: massive hemorrhage, suspected neoplasia, and mistake diagnosis; **it was not possible to recover data related to the indication for surgical treatment. Source: Elaborated by the authors.

**Table 2:** Comparison between demographic and clinical characteristics of patients with Crohn’s disease in relation to the occurrence of the surgical resection.

Characteristics and clinical variables	Surgical resection				*p*-value
	Present (n = 155)		Absent (n = 140)		
Duration of Crohn’s disease (mean ± standard deviation)	16.36 ± 9.58		10.54 ± 6.36		< 0.001
	**n**	**%**	**n**	**٪**	
**Gender**					**0.420**
Male	79	51	78	55.7	
Female	76	49	62	44.3	
**Smoking**					**< 0.001**
Yes	1	3.9	28	14.1	
No	25	96.1	179	85.9	
**Initial use of biologicals**					**< 0.001**
Yes	23	14.8	48	34.3	
No	132	85.2	92	65.7	
**Age at the time of inflammatory bowel disease diagnosis (years old)†**					**0.680**
< 16	14	9.1	17	12.1	
16–40	109	70.3	94	67.1	
> 40	32	20.6	29	20.8	
**Location of Crohn’s disease†**					
Ileal	34	21.9	46	32.9	
Colonic	6	3.9	28	20	**0.001**
Ileo-colonic	109	70.3	65	46.4	**0.003**
Upper gastrointestinal tract	6	3.9	1	0.7	
**Behavior of Crohn’s disease†**					
Inflammatory	4	2.6	57	40.7	
Stenosing	81	52.3	29	20.7	**< 0.001**
Fistulizing	70	45.2	54	38.6	**< 0.001**
Perianal disease	48	31	51	36.4	0.32
**Extraintestinal manifestation†**					**0.82**
Yes	18	11.6	15	10.7	
No	137	88.4	125	89.3	

† Involvement of other parts of the body, such as joints, eyes, skin, liver and bile ducts, in patients with inflammatory bowel diseases (ulcerative colitis and Crohn’s disease).

Source: Elaborated by the authors.

### Factors associated with surgical resection

The univariate analysis revealed the following results:

1. Smoking: smoking habit (active or former smoking) was significantly associated with surgical resection (p < 0.001; *odds ratio* – OR = 3; 95% confidence interval – 95%CI 1.76–5.12);
2. Biological agents: initial use of biological agents (defined as use up to one year after the diagnosis of CD) was significantly more frequent in the group of patients without surgical resection (34.3% vs. 14.8%; *p* < 0.001; OR = 0.33; 95%CI 0.19–0.59) (Fig. 2);
  **Figure 2:**
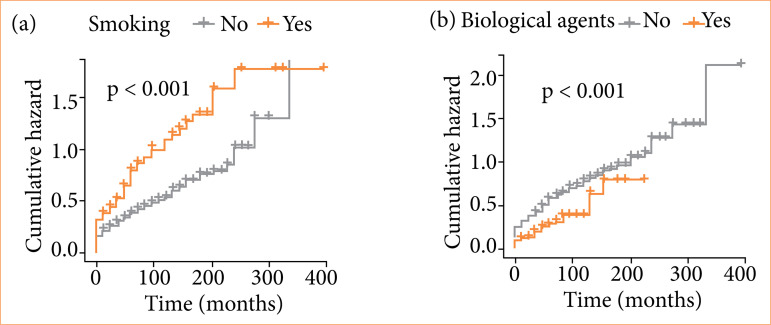
Analysis of the risk of surgical resection in Crohn’s disease patients using Kaplan–Meier curves stratified by predictive factors. **(a)** Cumulative risk of surgical resection was significantly higher in smoking patients. **(b)** Cumulative risk of surgical resection was significantly higher in patients without initial use of biological agents.

Source: Elaborated by the authors.
3. CD diagnosis time: the mean disease time was significantly longer in the group of patients with surgical resection than those without surgical resection (16.36 ± 9.58 years vs. 10.54 ± 6.36 years; *p* < 0.001);
4. Location of CD: ileo-colonic location (L3) was significantly associated with surgical resection (70.3% vs. 46.4%; *p* = 0.003; OR = 2.27; 95%CI 1.32–3.89) when compared to those with ileal location (L1). On the other hand, the frequency of colonic location (L2) was significantly higher in the group without surgical resection when compared to those with the ileal location (L1) [20% *vs*. 3.9%; p = 0.01; OR = 0.29; 95%CI 0.11–0.78];
5. Behavior of CD: stenosing phenotype (B2) was significantly associated with surgical resection (53.3% vs. 2.6%; *p* < 0.001; OR = 39.80; 95%CI 13.26–119.43) when compared to the inflammatory phenotype (B1). Similarly, the fistulizing phenotype (B3) was also significantly associated with surgical resection (45.2% *vs*. 2.6%; *p* < 0.001; OR = 18.47; 95%CI 6.31–54.08) (45.2% vs. 2.6%; *p* < 0.001; OR = 18.47; 95%CI 6.31–54.08) when compared to the inflammatory phenotype (B1) (Fig. 3);
  **Figure 3:**
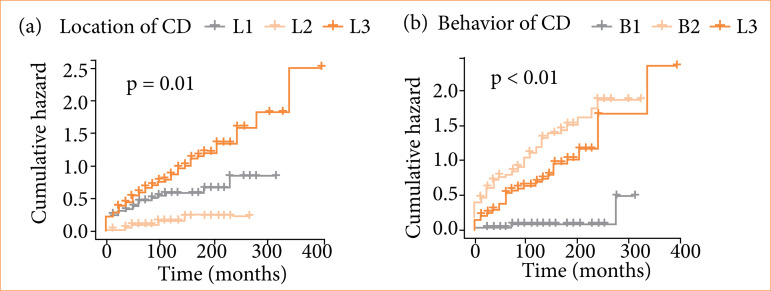
Analysis of the risk of surgical resection in Crohn’s disease (CD) patients using Kaplan–Meier curves stratified by predictive factors. **(a)** Cumulative risk of surgical resection was significantly higher in ileo-colonic (L3) and colonic (L2) locations when compared to ileal location (LI). **(b)** Cumulative risk of surgical resection was significantly higher in stenosing (B2) and fistulizing (B3) phenotypes when compared to inflammatory phenotype (B1).

Source: Elaborated by the authors.
6. No associations with surgical resection were observed when considering the following baseline characteristics: gender, age at diagnosis (< 17, 17–40, > 40 years old), ethnicity, and presence of EIM (*p* > 0.05).

In the multivariate analysis, we found the following independent risk factors for surgical resection, as shown in Table 3:

**Table 3:** Associated factors with surgical resection among Crohn’s disease patients.

Clinical variables	Univariate analyses				Multivariate analyses		
	OR	95%CI	*p*-value		OR	95%CI	*p*-value
Smoking	3	1.76–5.12	< 0.001		3.30	1.67–6.51	0.001
Initial use of biologicals	0.33	0.19–0.59	< 0.001		-	-	-
Longer diagnosis of Crohn’s disease	-	-	< 0.001		1.08	1.04–1.12	< 0.001
Ileo-colonic location	2.77	1.32–3.89	0.003		1.88	0.99–3.83	0.008
Colonic location	0.29	0.11–0.78	0.01		-	-	-
Stenosing phenotype	39.80	13.26–119.43	< 0.001		39.79	10.07–106.82	< 0.001
Fistulizing phenotype	18.47	6.31–54.08	< 0.001		16.48	5.18–52.44	< 0.001

OR: odds ratio; 95%CI: 95% confidence interval. Source: Elaborated by the authors.

- smoking (OR = 3.30; 95%CI 1.67–6.51; *p* = 0.001);
- longer disease time (OR = 1.08; 95%CI 1.04–1.12; *p* < 0.001);
- ileo-colonic location (L3) [OR = 1.88; 95%CI 0.99–3.83; *p* = 0.008];
- stenosing phenotype (B2) [OR = 39.79; 95%CI 10.07–106.82; *p* < 0.001];
- fistulizing phenotype (B3) [OR = 16.48; 95%CI 5.18–52.44; *p* < 0.001].

## Discussion

In this study of a single reference center in Southeastern Brazil, we observed the need for surgical resection in 155 (52.5%) CD patients during an average follow-up of 137.36 ± 86.63 months. Smoking, longer disease duration, ileo-colonic location, and stenosing and fistulizing phenotypes were associated with a higher risk of surgical resection in patients with CD in our series. Surgical treatment for CD is generally reserved for cases of failure of medical treatment or complications, such as strictures resulting in intestinal obstruction, fistulas with associated abscess, hemorrhage, and suspected neoplasia or malignant transformation^10^^,^ 15 ^–^ 17.

Intestinal obstruction from stenosis is a frequent complication and currently the main indication for surgery in CD^10^^,^ 11. Acute obstruction can occur due to a primary stenosis or a series of strictures, which probably result from active inflammation and often resolve with medical treatment^10^^,^ 11 ^,^ 15. On the other hand, chronic obstruction, which usually results from a fixed fibrostenotic lesion, tends to require surgical treatment and commonly involves resection of the diseased segment^10^^–^ 12. In our study, stenosis with intestinal obstruction was the most common indication for surgical treatment and was observed in 69 (44.5%) patients with CD.

Failure of medical therapy is defined as failure to achieve sufficient response with the presence of symptoms that cannot be controlled with optimized medical therapy (refractory disease), treatment side effects, and inability of the patient to maintain compliance10. Currently, the failure of medical treatment still represents an important indication for surgery in some series of patients with CD (mainly of the small intestine)^10^^,^ 12. In our series, there were 24 (15.5%) cases of failure of medical treatment, including optimization of doses and changes in biological agents, maximum doses of immunosuppressants, and steroid dependence.

Fistulas with associated abscess or stenosis represent other common complications of CD that require surgery^15^^,^ 17. Approximately one-third of patients with CD develop intra-abdominal fistulas during their disease^1^^,^ 2. Intra-abdominal fistulas were responsible for 24 (15.5%) cases of indications for surgery in patients with CD in our series.

Another indication for surgical treatment of CD is EIM, which can occur in up to 25% of patients^1^^,^ 2 ^,^ 18. In general, they are associated with the presence of perianal disease and colonic disease. In our series, there was no association between the presence of EIM and the occurrence of surgical resection. A possible explanation for this result may be that greater use of biological and immunosuppressive therapy in this group of patients may contribute to a decrease in intestinal resection rates in CD patients.

Smoking represents the most well-characterized environmental factor in IBD, and smokers are at higher risk of developing CD and less risk of developing ulcerative colitis^19^^–^ 21. Furthermore, smokers with CD tend to have a more severe disease course with increased relapse rates; higher need for corticosteroids, immunosuppressants, and biological agents; higher rates of postoperative recurrence; complications such as stenosis; and greater need for hospitalization^19^^–^ 21.

However, in relation to the association between smoking and the need for surgical resection in patients with CD, studies that specifically assess this aspect have not reached a consensus^21^^–^ 24. Some have shown that smoking increases the risk of surgical resection, while others have shown no association. Possible explanations for these heterogeneous results can be attributed to the different methodologies adopted between the studies and the particularities of the studied populations^22^^,^ 23. In agreement with most studies, we observed that both current and former smokers were associated with a higher risk of surgical resection in patients with CD.

We observed an association between initial use of biological agents and lower rates of surgical resection in CD patients. The advent of biological therapy with the introduction of anti-TNF-alpha agents has represented a major advance in the clinical treatment of patients with IBD^25^. However, their role in reducing surgery rates remains elusive. In the early 2000s, the first randomized clinical trials of biological therapy in patients with luminal CD (ACCENT I and CHARM) demonstrated very low rates of surgical interventions, which ranged from 0.6 to 3% in one year in groups of patients treated with biological therapy^25^^,^ 26. However, these low rates of surgical resection in the initial randomized studies have not been reproduced in studies of the general population^27^^,^ 28.

Population-based cohort studies in patients with IBD (Denmark, Wales, Olmsted County) indicated that, in 30 years from diagnosis, cumulative risks were approximately 60% for surgery (defined as intestinal resection, not perianal surgery)^28^^–^ 30. However, it is important to note that the data from these studies represent indirect evidence that biological agents played a role in decreasing surgery over time because many of the patients included in these cohorts did not use biological agents as a clinical treatment option^28^^,^ 30. On the other hand, another study evaluated 296 patients treated at the University of Nancy Hospital in France between 2000 and 2008 (all diagnosed after 2000). The study found that, among patients who underwent surgery, 60% had been treated with at least one biological agent^31^. Thus, there was a decline in the rates of surgical resection in a patient with CD over time, but not substantially.

The need for intestinal resection in patients with CD increases with the duration of the disease. Approximately half of CD patients will undergo at least one surgery within 10 years of diagnosis^1^^,^ 2. A systematic review and meta-analysis by Frolkis et al.^29^ demonstrated that the need for intestinal resection in patients with CD increases from 16% in the first year after diagnosis to almost 50% within a decade after diagnosis^30^. Similar results were observed in two population-based Scandinavian studies^28^^,^ 32.

Our data agree with previous studies in that we observed association between higher rates of intestinal resection and longer duration of CD.

In our study, ileocolic location (L3), stenosing (B2), and penetrating (B3) disease behavior at diagnosis were independent risk factors for subsequent bowel surgery resection. Previous studies have assessed the relationship between disease location and behavior according to the Vienna and Montreal Classification at diagnosis and the risk of surgery resection^12^^,^ 31. Initial penetrating behavior and ileal location were risk factors for surgery in a recent study from Hungary^33^. On the other hand, no significant correlation was found between behavior at diagnosis and the risk of surgical resection in a Europe-wide population-based study^34^.

Our study provides a comprehensive summary of the factors associated with the occurrence of surgical resection in patients with CD, but it has some limitations that deserve to be highlighted. First, this was a study that included patients followed-up at a single reference university hospital. Secondly, our sample size was relatively small. Thirdly, a percentage of patients underwent surgery in other health institutions at the time of diagnosis, making it difficult to accurately specify the duration of the disease, as well as the treatments instituted and finally the absence of information regarding the nutritional status of the patients.

The factors associated with a higher occurrence of intestinal resection in patients with CD have already been well characterized in previous studies. However, most of these data came from North America and Europe. Consequently, there is a scarcity of data regarding factors associated with CD surgery in other geographic environments. This makes our study quite pertinent, and it may be important given the potential for immunogenic differences and differential management paradigms in different populations worldwide.

## Conclusion

A significant percentage of patients with CD will still need surgical treatment. In our series, the presence of stenosis, intra-abdominal fistulas, and failure to medical treatment were the most common indications for surgical treatment. Smoking habits, longer disease duration, ileo-colonic location, and stenosing and fistulizing phenotypes at diagnosis were the main factors associated with a higher risk of surgical resection.

Despite its limitations, this study has described the experience of an important IBD reference center in Southeastern Brazil. The study provides important information on the rate and factors associated with surgical resection in patients with CD. Adequate initial clinical treatment, multidisciplinary approaches, and strict monitoring are strategies that must be adopted in the management of these patients while aiming to identify factors associated with a higher risk of surgical resection.

## Data Availability

All data sets were generated or analyzed in the current study.
